# Meta-analysis of post-transcatheter aortic valve replacement outcomes in patients with cardiac amyloidosis and aortic stenosis

**DOI:** 10.1097/JS9.0000000000000532

**Published:** 2023-06-07

**Authors:** Vikash Jaiswal, Amey Joshi, Angela Ishak, Megha Nataraj, Song P. Ang, Nida Khan, Farshid Daneshvar, Victor H. Aguilera-Alvarez, Deepak Verma, Abhigan B. Shrestha, Prachi Sharma

**Affiliations:** a JCCR Cardiology Research, Varanasi, India; b Vydehi Institute of Medical Sciences and Research Centre, Bangalore, India; c European University Cyprus, School of Medicine, Nicosia, Cyprus; d Cardio-Pulmonary Physiotherapist and Research, Manipal, Karnataka, India; e Division of Internal Medicine, Rutgers Health/Community Medical Center, New Jersey, USA; f Jinnah Sindh Medical University, Karachi, Pakistan; g Department of Cardiology, AdventHealth, FL, USA; h Universidad Autónoma de Baja, California, USA; i Janaki Medical College, Dhanusha, Nepal; j Department of Internal Medicine, M Abdur Rahim Medical College, Dinajpur, Bangladesh; k Division of Cardiology, Department of Internal Medicine, King George's Medical University, India

*Dear Editor*,

The prevalence of cardiac amyloidosis (CA) has been grossly underestimated due to the lack of specific screening guidelines and phenotypic overlap with other cardiac diseases. However, recent advances in diagnostic techniques and pharmacotherapies have helped significantly improve CA outcomes^1^. Aortic stenosis (AS) shares several common features with CA, and recent evidence suggests the concomitance of these pathologies^2^. Without appropriate and timely intervention, severe AS and CA can individually have a poor prognosis and, if in coexistence, delay diagnosis and timely treatment^3^. This systematic review and meta-analysis provide insights into the outcomes of transcatheter aortic valve replacement (TAVR) in patients with severe AS and concomitant CA. This study aims to clarify the potential benefits of aortic valve replacement in this high-risk population and its approach.

A comprehensive search was conducted on PubMed, Embase, and Scopus utilizing predefined MeSH (Medical Subject Headings) terms coupled with Boolean operators ‘AND’ and ‘OR’. The search strategy included the terms ‘Cardiac Amyloidosis’ OR ‘Amyloidosis’ AND ‘Aortic Stenosis’ AND ‘TAVR’ OR ‘Aortic valve replacement’ AND ‘Outcomes’. Our meta-analysis sought observational studies published from inception up until 10th April 2023. Studies with patients of age at least 18 years, two groups of patients in which one group is cardiac amyloidosis with aortic stenosis (CA+AS), and another group of patients with aortic stenosis alone (AS). Baseline continuous variables were summarized as mean (SD), whereas dichotomous variables were described as frequencies or percentages. A conventional, two-arm meta-analysis for primary and secondary outcomes was performed by adopting the Dersimonian and Laird random-effects model for the study variations. Outcomes were reported as pooled odds ratio (OR) and their corresponding 95% confidence interval (95% CI). Statistical significance was met if 95% CI did not cross the numeric ‘1’ and the two-tailed *P* value was less than 0.05. The heterogeneity among studies was assessed using the Higgins *I*-squared (*I* ^2^) statistical model, with *I* ^2^ values less than 75% considered mild-moderate and at least 75% considered high.

We identified four studies^4–7^ with a total of 246 151 patients who underwent TAVR. Among these, 586 individuals had CA+AS, while 245 565 has AS alone. The mean age of patients among CA+AS and AS-alone groups were 85 and 80 years, respectively. The most common comorbidity present among the CA+AS group when compared to AS-alone was hypertension (45.05 vs. 0.2%).

The pooled analysis of primary endpoints showed the odds of post-TAVR short-term mortality within 30 days [OR, 0.34 (95% CI: 0.10–1.19), *P*=0.09, *I* ^2^=62.40%] was comparable between both groups of patients. However, the incidence of stroke [OR, 0.34 (95% CI: 0.18–0.65), *P*<0.001, *I* ^2^=16.78%], acute kidney injury (AKI) [OR, 0.50 (95% CI: 0.34–0.73], *P*<0.001, *I* ^2^=32.09%] were significantly lower in AS group compared with CA+AS group. Pooled odds of major bleeding were comparable between both groups of patients [OR, 0.88 (95% CI: 0.54–1.44), *P*=0.62, *I* ^2^=32.95%] (Fig. 1A–D).

**Figure 1:**
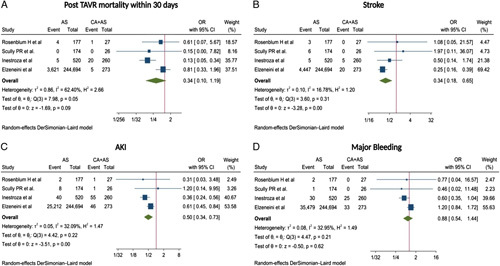
Forest plot of clinical outcomes among meta-analysis: (A) post-TAVR mortality within 30 days, (B) stroke, (C) AKI, and (D) major bleeding. AKI, acute kidney injury; AS, aortic stenosis; CA, cardiac amyloidosis; OR, odds ratio; TAVR, transcatheter aortic valve replacement

To our knowledge, the present meta-analysis is the first to evaluate clinical outcomes post-TAVR among those with CA and AS. It is expected for patients with dual pathology (CA+AS) to have worse outcomes post-TAVR as they usually have an increase in risk factors such as old age, elevated troponin levels, history of carpal tunnel syndrome, increased septal thickness on echocardiography, and right bundle branch block on electrocardiogram. Previous studies have stipulated that due to these factors, there is an increase in 1-year mortality among patients with (CA+AS)^8^. However, our analysis showed that short-term mortality was comparable among both groups. Additionally, recent studies have shown that there was no difference in all-cause mortality in the patients who received TAVR, despite a more severe clinical picture in dual pathology (CA+AS) (grade 2/3), with higher biomarkers, reduced functional capacity, NYHA (New York Heart Association) class 3 or 4, and altered ventricular function^8^.

Interestingly, this analysis showed that major bleeding and short-term mortality were comparable between both patient populations post-TAVR, as previous reports showed there was an increase in the post-SAVR^9^. This means that patients with amyloidosis if they have AS or otherwise, could benefit from less invasive treatment options like TAVR. A recent study also showed that TAVR showed lower in-hospital mortality in patients with CS than SAVR^10^. However, studies comparing the outcome of patients with CS and AS post-TAVR and SAVR would be required.

The major limitation of the current study is that the results were largely derived from observational studies, where confounding bias could not be ruled out. Secondly, only four studies are available to date to evaluate the outcomes, and hence these results cannot be generalized.

Our results show that mortality was comparable between both groups of patients, while the incidence of stroke and AKI was significantly lower in AS group compared with the CA+AS group.

## Ethical approval

Not applicable.

## Consent

Not applicable.

## Source of funding

Not applicable.

## Author contribution

V.J.: conceptualization; V.J., A.J., A.I., M.N., S.P.A., N.K., F.D., V.H.A.-A., D.V., A.B.S., and P.S.: manuscript writing and editing. All the authors have reviewed the manuscript.

## Conflicts of interest disclosure

There are no conflicts of interest.

## Guarantor

Vikash Jaiswal.

## Data availability statement

Data will be available on request from the corresponding author.

## Provenance and peer review

Not commissioned, externally peer-reviewed.

## Disclosure

The abstract of this study has been accepted and presented at the AHA22 conference.
